# A Smart pH-Sensitive Delivery System for Enhanced Anticancer Efficacy via Paclitaxel Endosomal Escape

**DOI:** 10.3389/fphar.2019.00010

**Published:** 2019-01-24

**Authors:** Yihua Yang, Zhe Wang, Ying Peng, Jinsong Ding, Wenhu Zhou

**Affiliations:** ^1^Xiangya School of Pharmaceutical Sciences, Central South University, Changsha, China; ^2^Jiangsu Key Laboratory of New Drug Research and Clinical Pharmacy, School of Pharmaceutical Sciences, Xuzhou Medical University, Xuzhou, China; ^3^Xiangya International Academy of Translational Medicine, Central South University, Changsha, China

**Keywords:** pH-sensitive, micelles, cancer, paclitaxel, endosomal escape

## Abstract

Micelles are highly attractive nano-drug delivery systems for targeted cancer therapy. While they have been demonstrated to significantly alleviate the side-effects of their cargo drugs, the therapy outcomes are usually suboptimal partially due to ineffective drug release and endosome entrapment. Stimulus-responsive nanoparticles have allowed controlled drug release in a smart fashion, and we want to use this concept to design novel micelles. Herein, we reported pH-sensitive paclitaxel (PTX)-loaded poly (ethylene glycol)-phenylhydrazone-dilaurate (PEG-BHyd-dC_12_) micelles (PEG-BHyd-dC_12_/PTX). The micelles were spherical, with an average particle size of ∼135 nm and a uniform size distribution. The pH-responsive properties of the micelles were certified by both colloidal stability and drug release profile, where the particle size was strikingly increased accompanied by faster drug release as pH decreased from 7.4 to 5.5. As a result, the micelles exhibited much stronger cytotoxicity than the pH-insensitive counterpart micelles against various types of cancer cells due to the hydrolysis of the building block polymers and subsequent rapid PTX release. Overall, these results demonstrate that the PEG-BHyd-dC_12_ micelle is a promising drug delivery system for cancer therapy.

## Introduction

With the development of nanotechnology, various materials such as polymers, lipid, and metals (oxides), have been widely applied to design drug delivery system, especially for cancer therapy (Farokhzad and Langer, 2009). Nanoparticles based on the above materials have been demonstrated to realize controlled drug release and effectively targeting drug delivery (Wilczewska et al., 2012). To this end, micelles composed of amphipathic copolymers have received wide attention owing to their attractive features, such as small and uniform size, tumor targeting ability *via* the enhanced permeability and retention (EPR) effect, high stability in aqueous solution and excellent biocompatibility (Felber et al., 2012; Liu J. et al., 2014; Wang et al., 2018).

However, albeit with the extensive research efforts, the clinical translations of micelles from bench to bedsides are rather limited, partially due to their suboptimal therapy outcomes caused by the inefficient drug release at the tumor site and the endosomal entrapment of micelles (Kanamala et al., 2016). Plain micelles exhibit relatively slow drug release rate, which may result in ineffective drug concentration inside targeted cells (Wu et al., 2013). To mitigate these issues, smarter micelles are desired to be equipped with endosomal escape and rapid drug release abilities, which could be able to provide sufficient drug concentration for effective killing of the tumor cells.

To achieve such goals, environmentally sensitive polymers that can respond to different stimuli to trigger drug release have been extensively investigated, such as light (Liu et al., 2012; Cao et al., 2013), temperature (Kim et al., 2010; Wang et al., 2014), ultrasound (Yin et al., 2013; Ahmed et al., 2015), magnetic field (Ao et al., 2014; Deng et al., 2015), pH (Liu Y. et al., 2014; Yuba et al., 2017), redox properties (Yin et al., 2015; Zhang et al., 2016), and enzyme activity (Rao and Khan, 2013; Harnoy et al., 2014). Among of them, the pH-sensitive polymeric micelle appears to be a highly appealing candidate due to the intrinsic differences between solid tumors and the surrounding normal tissues in terms of their relative acidity. The pH-sensitive polymer micelles were devised based on copolymers composed of hydrophobic and hydrophilic polymers linked *via* acid-liable bonds, including hydrazone (Mo et al., 2012), benzoic imine (Yuan et al., 2012), oxime (Liu B. et al., 2014), acetal (Li et al., 2016), ester (Gao et al., 2018) and orthoester (Tang et al., 2011). Hydrolysis of the acid-labile bonds leads to rapid drug release at an acidic pH.

Herein, we synthesized the amphiphilic polymer PEG-BHyd-dC_12_ via an acid-labile hydrazone bond and constructed pH-responsive micelles. The hydrophilic PEG segment on micelles surface affords high colloidal stability *in vitro* and long circulation time *in vivo*, while it is readily departed from micelles at the tumor site under acid conditions, which is beneficial for cellular uptake (Du et al., 2011). Paclitaxel (PTX), one of the most effective antitumor drugs, was encapsulated into micelles due to its hydrophobic nature, and released in a pH-responsive manner. For comparison, the pH-insensitive counterpart polymer of PEG-BAmi-dC_12_ was also synthesized for micelles preparation. The physicochemical characterization, colloidal stability, drug release, cellular uptake, and *in vitro* cytotoxicity of the micelles were evaluated.

## Materials and Methods

### Chemicals and Reagents

Paclitaxel (PTX), 1-ethyl-3-[3-dimethylaminopropyl] carbodiimide hydrochloride (EDC), *N*-hydroxysulfosuccinimide (NHS), 4-dimethylaminopyridine (DMAP), lauroyl chloride, α-methoxy-x-amino-poly(ethylene glycol) (Mn = 2000) (MeO-PEG2000-NH_2_) were purchased from Shanghai Aladdin Reagent Co. Ltd. (Shanghai, China). mPEG-hydrazide (Mn = 2000) was from Seebio Biotech, Inc. (Shanghai, China), and 3,5-dihydroxybenzaldehyde was from Bide Pharmatech Ltd. (Shanghai, China). 3,5-Dihydroxybenzoic acid was obtained from Saen Chemical Technology Co. Ltd. (Shanghai, China). Potassium hydroxide (KOH), tetrahydrofuran (THF), dimethyl sulfoxide (DMSO), petroleum ether, ethyl acetate were purchased from Sinopharm Chemical Reagent Co., Ltd. (Shanghai, China). 3-(4,5-dimethylthiazol-2-yl)-2,5-diphenyl tetrazolium bromide (MTT), coumarine (Cou-6) and 4′,6-diamidino-2-phenylindole (DAPI) were obtained from Sigma-Aldrich Co. (St. Louis, MO, United States). Lysotracker red was supplied from Beyotime Institute of Biotechnology (Jiangsu, China). Dulbecco’s modified Eagle’s medium (DMEM), RPMI 1640, penicillin, streptomycin, phosphate buffered saline (PBS), fetal bovine serum (FBS) were purchased from Gibco Life Technologies, Inc. (Carlsbad, CA, United States). Human lung cancer cells (A549), human breast cancer cells (MDA-MB-231), human ovarian cancer cells (A2780) were obtained from Xiangya cell center (Changsha, China). PTX-resistant human lung cancer cells (A549/T) was bought from Gefan Biotechnology Co., Ltd. (Shanghai, China).

### Synthesis of the pH-Sensitive Copolymer PEG-BHyd-dC_12_

3,5-Dihydroxybenzaldehyde was dissolved in THF, followed by the addition of KOH. Lauroyl chloride was added dropwise into the above mixture and vigorously stirred for 6 h to yield 3,5-dilaurate benzaldehyde. The purified 3,5-dilaurate benzaldehyde and mPEG-hydrazide were dissolved in ethyl alcohol and stirred for 24 h. After purification, the final amphiphilic polymer PEG-BHyd-dC_12_ was obtained.

### Synthesis of the pH-Insensitive Copolymer PEG-BAmi-dC_12_

First, lauroyl chloride was added dropwise to a mixture of 3,5-dihydroxybenzoic acid with KOH in anhydrous acetone at 0°C under stirring to obtain 3,5-dilaurate benzoic acid. Then, 3,5-dilaurate benzoic acid, EDC, DMAP and NHS were dissolved into DMSO and stirred at room temperature for 2 h, followed by the addition of MeO-PEG2000-NH_2_. The resulting solution was dialyzed and subsequently lyophilized to obtain PEG-BAmi-dC_12_.

### Characterization of Copolymers

The ^1^H-NMR spectra of PEG-BHyd-dC_12_ and PEG-BAmi-dC_12_ were recorded using a Bruker Avance 400 MHz NMR spectrometer (Varian, United States) with deuterated chloroform (CDCl_3_) as the solvent. The self-assembly behavior of polymers was investigated by the fluorescence probe technique (Xiong et al., 2017). First, 100 μL of pyrene in acetone (2.9 × 10^−2^mM) was evaporated to form a thin film on the flask bottom. Then, various concentrations of polymer solutions (from 0.1 μg/mL to 200 μg/mL) were added to the pyrene-coated vials and stored in the dark overnight. The fluorescence intensity ratio of I_337_/I_334_ in the emission spectra of pyrene was calculated and plotted against the logarithm of the polymer concentrations. The CMC value was obtained based on the fluorescence excitation spectra of the mixed solution.

### Preparation of Micelles

PTX-loaded micelles were prepared by a thin-film hydration method. In brief, PEG-BHyd-dC_12_ or PEG-BAmi-dC_12_ (20.0 mg) and PTX (1 mg) were dissolved in dichloromethane (4 mL). The solution was evaporated under reduced pressure to form a uniform film. Deionized water (10 mL) was added and rotated for another 1 h. The obtained colloidal solution was then centrifuged at 3,000 rpm for 10 min and filtered through 0.45 μm pore size filter, followed by lyophilization. Blank micelles were prepared in a similar way in the absence of PTX.

### Characterization of Micelles

The particle size, PDI, and zeta potential measurement were determined by dynamic light scattering (DLS) method using a Malvern Zeta Sizer Nano series (Nano ZS, Malvern Instruments, United Kingdom) at 25°C. The morphologies of the micelles were observed using transmission electron microscopy (TEM) (Titan G2-F20, FEI, United States).

The determination of PTX was carried out using a high-performance liquid chromatography (HPLC) system (LC-2010, Shimadzu, Tokyo, Japan). The chromatographic column was an ODS C_18_ (250 × 4.6 mm, 5 μm, Diamonsil, Beijing, China). The mobile phase consisted of mixtures of acetonitrile and water (55:45, v/v). The flow rate was 1 mL⋅min^−1^, and the detection wavelength was 227 nm. Micelles were centrifuged in an ultrafiltration tube (MWCO 10 kDa) at 5,000 rpm for 10 min and filtered through 0.22 μm filter to remove the unloaded PTX. PTX-loaded micelles were disrupted by methanol. The PTX loading content (LC) and encapsulated efficiency (EE) were calculated using the following formulae:

$$\begin{array}{lll} \mathrm{EE}\;(\%) & = & \mathrm{Amount of PTX in micelles}/\\ & & \;\mathrm{Amount of PTX fed initially}\times 100\%\\ \mathrm{LC}\;(\%) & = & \mathrm{Amount of PTX in micelles}/\\ & & \;\mathrm{Amount of PTX-loaded micelles}\times 100\% \end{array}$$

### Colloidal Stability

Micelles were incubated with 10% FBS or 10 mM phosphate buffer solutions (pH 7.4, 6.5, and 5.5) at 37°C for 72 h, and the size was measured by DLS at different intervals.

### *In vitro* Drug Release

The release study was assessed by the dialysis method. The release media was PBS solutions containing 0.5% Tween-80 with different pH values (5.5, 6.5, and 7.4). Typically, 2 mL of PTX-loaded micelles was placed in a dialysis bag (MWCO 3500) and dialyzed against 25 mL of buffer medium under mechanical shaking (100 rpm) at 37°C. At predetermined time intervals, 2 mL of release medium was withdrawn and replenished with an equal volume of fresh medium. The released PTX was detected by HPLC.

### Cell Culture

A549 and A549/T cells were maintained in RPMI 1640 medium supplemented with 10% FBS, penicillin (50 U/mL) and streptomycin (50 U/mL) in a 5% CO_2_ atmosphere at 37°C. MDA-MB-231 and A2780 were maintained in DMEM medium supplemented with 10% FBS, penicillin (50 U/mL) and streptomycin (50 U/mL) in a 5% CO_2_ atmosphere at 37°C.

### Intracellular Distribution

Cou-6 loaded micelles were constructed according to the above method, except the drug was replaced with Cou-6. A549 cells were seeded on glass coverslips in the 24-well plates at a density of 4 × 10^4^ per well. After culturing for 24 h, Cou-6 loaded micelles ([Cou-6] = 200 ng/mL) were added and incubated for 1 h. Alternatively, the cells were incubated with Cou-6 loaded micelles for 1 h, then washed and cultured in fresh media for another 3 h. Then, the medium was replaced with 70 nM lysotracker red and incubated for another 1 h. Afterward, the cells were fixed with 4% formaldehyde for 20 min at room temperature and visualized using a CLSM (LSM 780, Carl Zeiss, Jena, German).

### Cellular Uptake

A549 cells were seeded in 6-well plates with a density of 3 × 10^5^ cells per well and incubated overnight, and then, the medium was replaced with Cou-6 loaded micelles at final Cou-6 concentration of 200 ng/mL. After 1 h or 4 h of incubation, the cells were harvested and quantified by flow cytometry (FACSVerse, BD, United States).

### Cytotoxicity Assay

The cytotoxicity of micelles with or without an anticancer drug was determined by MTT assay. The cells were seeded in a 96-well plate at a density of 6,000 cells per well and maintained for 24 h. The medium was then replaced with the micelles and further incubated for 72 h. Then, 20 μL of MTT solution (5 mg/mL) was added to each well of the plate for another 4 h. Subsequently, 100 μL of DMSO was added to dissolve the formazan crystals, and the absorbance was measured at 570 nm by a microplate reader (ELX800, Bio-Tek, United States). The untreated cells were used as controls.

### Hemolysis Tests

The hemocompatibility of micelles was evaluated by hemolysis assay (Yang et al., 2016). First, fresh rabbit blood was extracted from the heart of a rabbit. Subsequently, erythrocytes were obtained by centrifugation at 3,000 rpm for 15 min and washed with normal saline (NS). Serial dilutions of micelles were then added to the 2% erythrocytes (v/v) and incubated for 2 h at 37°C in a thermostatic water bath. Finally, the mixtures were centrifuged at 3,500 rpm for 15 min, and the supernatant of all samples was measured for UV absorbance (A) at 540 nm. NS and 0.5% Triton X-100 were regarded as the negative and positive controls, respectively. The hemolysis ratio was calculated as follows:

$$\begin{array}{lll} \text{Hemolysis }(\%) & = & ({\text{A}}_{\text{sample}}-{\text{A}}_{\text{control}(-)})/\\ & & \left({\text{A}}_{\text{control}(+)}-{\text{A}}_{\text{control}(-)}\right) \end{array}$$

### Statistical Analysis

The data were expressed as the mean ± standard deviation (SD). Statistical analysis was performed using a two-tailed Student’s *t*-test and analysis of variance (ANOVA) with the aid of SPSS 23.0 software. Differences were considered statistically significant when *p-*values were less than 0.05.

## Results and Discussion

### Synthesis and Characterizations of Copolymers

**FIGURE 1:**
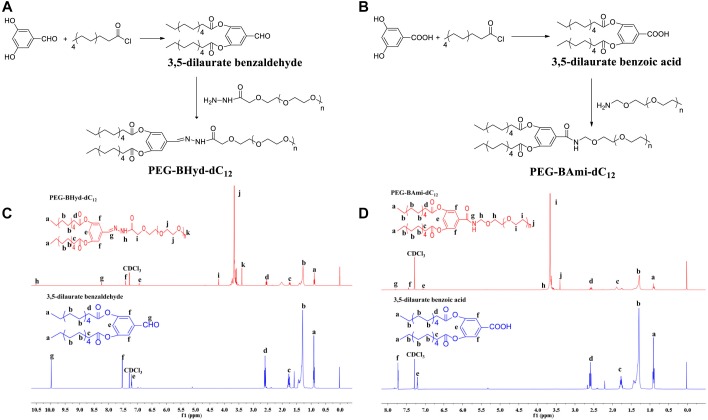
Synthesis and characterization of PEG-BHyd-dC_12_ **(A,C)** and PEG-BAmi-dC_12_ **(B,D)**_._ The synthesis of PEG-BHyd-dC_12_ **(A)** and PEG-BAmi-dC_12_ **(B)**, H1-NMR spectrum of PEG-BHyd-dC_12_ **(C)**, and PEG-BAmi-dC_12_ **(D)**.

The synthesis schemes of PEG-BHyd-dC_12_ and PEG-BAmi-dC_12_ were illustrated in Figures 1A,B. To synthesize the PEG-BHyd-dC_12_ di-block amphiphilic polymer, the hydrophobic fragment of 3,5-dilaurate benzaldehyde was conjugated with the hydrophilic fragment of PEG through the linkage of hydrazone. The 3,5-dihydroxybenzaldehyde was first reacted with lauroyl chloride to form a 3,5-dilaurate benzaldehyde intermediate with a yield of 90%, and then the aldehyde group on 3,5-dilaurate benzaldehyde reacted with the hydrazine groups on mPEG-hydrazide to give PEG-BHyd-dC_12_ with a final yield was 69%. All of the synthetic compounds were characterized by ^1^H-NMR spectra (Figures 1C,D), which were in good agreement with their depicted structures as described in the following:

^1^H NMR of intermediate compound 3,5-dilaurate benzaldehyde: ^1^H NMR (400 MHz, CDCl_3_) δ (ppm) 0.91 (6H, t, -CH_3_), 1.21–1.45 (32H, m, -(CH_2_)_n_), 1.75 (4H, m, CO-βH), 2,59 (4H, t, CO-αH), 7.20 (1H, t, 4-ArH), 7.52 (2H, d, 2,6-ArH), 9.98 (1H, s, -CHO).

^1^H NMR of PEG-BHyd-dC_12_: ^1^H NMR (400 MHz, CDCl_3_) δ (ppm) 0.89 (6H, t, -CH_3_), 1.22–1.45 (32H, m, -(CH_2_)_n_), 1.74 (4H, m, CO-βH), 2,54 (4H, t, CO-αH), 3.39 (3H, s, -OCH_3_ from PEG), 3.50–3.84 ((-OCH_2_CH_2_-)_n_), 4.19 (2H, s, CO-αH, from PEG), 6.94 (1H, t, 4-ArH), 7.41 (2H, d, 2,6-ArH), 8.24 (1H, s, -NH), 10.5 (1H, s, -CH = N).

As for PEG-BHyd-dC_12_, the characteristic peaks at 3.5–3.84 ppm were from PEG, and the proton peak at 10.5 ppm indicated the formation of the hydrazone bond. In addition, the absence of proton peak of aldehyde (9.98 ppm) suggested that free 3,5-dilaurate benzaldehyde was removed in the purified PEG-BHyd-dC_12_.

^1^H NMR of intermediate compound 3,5-dilaurate benzoic acid: ^1^H NMR (400 MHz, CDCl_3_) δ (ppm) 0.90 (6H, t, -CH_3_), 1.21–1.44 (32H, m, -(CH_2_)_n_), 1.71 (4H, m, CO-βH), 2,61 (4H, t, CO-αH), 7.20 (1H, t, 4-ArH), 7.72 (2H, d, 2,6-ArH).

^1^H NMR of PEG-BAmi-dC_12_: ^1^H NMR (400 MHz, CDCl_3_) δ (ppm) 0.90 (6H, t, -CH_3_), 1.22–1.45 (32H, m, -(CH_2_)_n_), 1.75 (4H, m, CO-βH), 2,58 (4H, t, CO-αH), 3.40 (3H, s, -OCH_3_ from PEG), 3.50–3.84 ((-OCH_2_CH_2_-)_n_), 7.06 (1H, t, 4-ArH), 7.45 (2H, d, 2, 6-ArH), 7.79 (1H, d, -CONH).

The characteristic peaks of PEG (3.50–3.84 ppm) were obvious, and the peak of new amide bond can be seen at 7.79 ppm for PEG-BAmi-dC_12_.

### CMC Measurement

**FIGURE 2:**
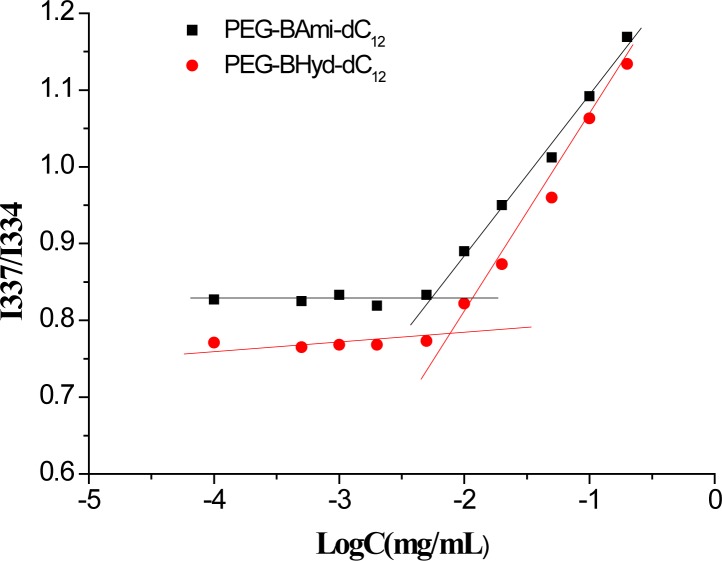
The CMC curve of PEG-BHyd-dC_12_ and PEG-BAmi-dC_12_.

As amphiphilic materials, a key parameter for their applications as a nanocarrier is their CMC. Micelles can be formed at concentrations above the CMC. The CMC values of PEG-BHyd-dC_12_ and PEG-BAmi-dC_12_ were determined by a well-established method using pyrene as a fluorescence probe, resulting in 7.5 μg/mL for PEG-BHyd-dC_12_ and 5.6 μg/mL for PEG-BAmi-dC_12_ (Figure 2). These CMC values were within the typical concentration range for most polymeric micelle CMCs, which can be directly applied *in vivo* (Maysinger et al., 2007; Diezi et al., 2010; Owen et al., 2012). It is reasonable that these two polymers have comparable CMC values, as their structures are nearly identical; they only differed at the junction between the hydrophobic and hydrophilic blocks (one with a hydrazone bond and the other with an amide bond). Therefore, PEG-BAmi-dC_12_ is an excellent control to study the pH-responsive property of PEG-BHyd-dC_12_ for drug delivery.

### Preparation and Characterization of Micelles

**FIGURE 3:**
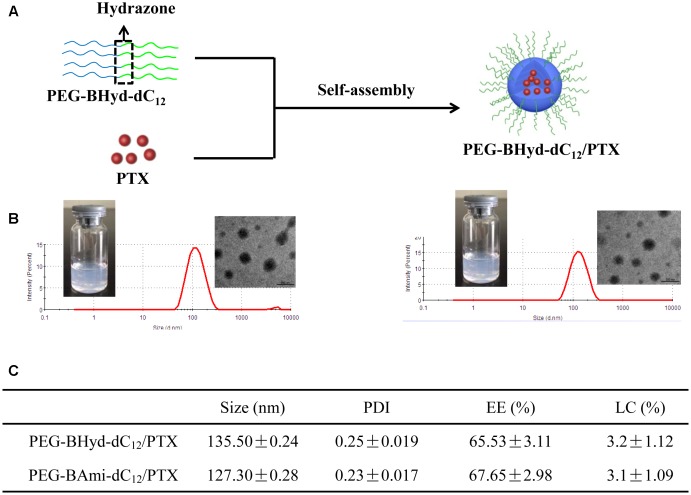
Preparation and characterization of micelles. Schematic preparation **(A)**, appearance, size distribution, and TEM images (**B**, left presents PEG-BHyd-dC_12_/PTX, right presents PEG-BAmi-dC_12_/PTX), characterization **(C)**. Data were presented as mean ± standard deviation (SD; *n* = 3).

From the above experiments, we have demonstrated that both PEG-BHyd-dC_12_ and PEG-BAmi-dC_12_ were able to self-assemble into micelles at very low concentrations, implying their applicability for the development of a nano-drug delivery system. We next used these polymers to prepare micelles, and the hydrophobic PTX was used a model to encapsulate into the hydrophobic core of the micelles (Figure 3A). The pH-sensitive micelles (PEG-BHyd-dC_12_/PTX) were prepared using a standard thin-film hydration method. After removing the organic solvents, the solution appeared to be semi-transparent with light-blue opalescence (Inset in Figure 3B, left), suggesting the successful preparation of nano-sized micelles. The particle size was approximately 135 nm as determined by DLS (Figure 3B, left); this size is suitable for passive accumulation in the tumor tissue through the EPR effect (Danhier et al., 2010). From TEM, the micelles were well dispersed with spherical morphology (Inset in Figure 3B, left). The LC efficiency of PEG-BHyd-dC_12_/PTX was 3% (Figure 3C), which was comparable to many other PTX-loading micelles reported previously, and was sufficient for subsequent *in vitro*/*in vivo* therapeutic applications (Lee et al., 2003; Zhu et al., 2010; Mei et al., 2015).

By using the same method, the pH-insensitive PEG-BAmi-dC_12_/PTX micelles were also prepared and characterized (Figure 3B, right; Figure 3C). Interestingly, these two types of micelles displayed quite similar properties in terms of appearance, particle size, morphology and drug loading efficiency. Therefore, a parallel comparison between these micelles can be made for their *in vitro/in vivo* biological performance, which can be rationalized by the pH-responsive bond linkage.

### Colloidal Stability

**FIGURE 4:**
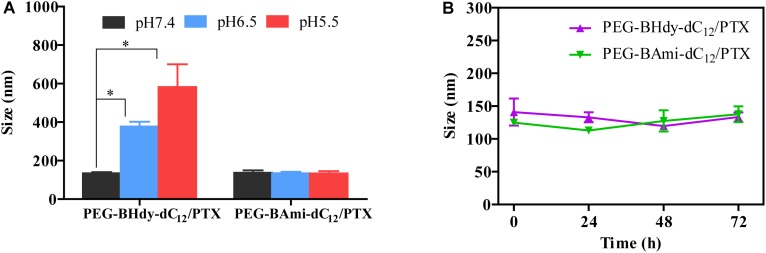
Colloidal stability of micelles. Size change of PEG-BHyd-dC_12_/PTX micelles and PEG-BAmi-dC12/PTX micelles in phosphate buffers with different pH values **(A)** and 10% FBS **(B)** at 37°C for 72 h. The pHs were buffered by disodium hydrogen phosphate and sodium dihydrogen phosphate with total phosphate concentration of 10 mM. Data were shown as mean ± SD (*n* = 3). ^∗^*p* < 0.05.

The colloidal stability of the micelles was first studied under different buffer solutions. Interestingly, with pH decrease from 7.4 to 6.5 and 5.5, the particle size of PEG-BHyd-dC_12_/PTX markedly increased, while it remained unchanged for PEG-BAmi-dC_12_/PTX (Figure 4A). This can be rationalized by the pH-responsive property of the PEG-BHyd-dC_12_/PTX, which could swell and then collapse at lower pH (Li et al., 2016; Qiu et al., 2017). We also challenged the micelles with 10% FBS, and both types of micelles were quite stable even after 72 h incubation (Figure 4B). Therefore, the pH-sensitive micelles were stable in blood circulation and can rapidly collapse to release the payload under acidic conditions.

### *In vitro* Drug Release

**FIGURE 5:**
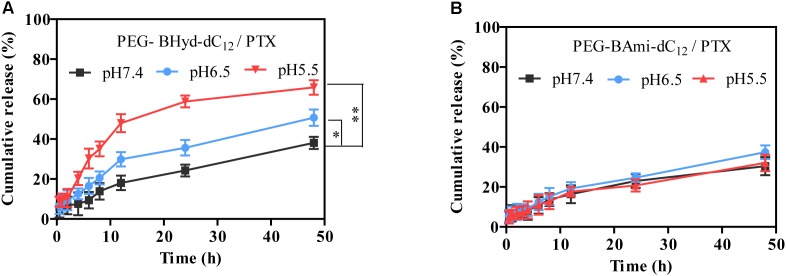
Release profiles of PEG-BHyd-dC_12_/PTX micelles **(A)** and PEG-BAmi-dC12/PTX micelles **(B)** at different pHs at 37°C. The pHs were buffered by disodium hydrogen phosphate and sodium dihydrogen phosphate with total phosphate concentration of 10 mM. Data were shown as mean ± SD (*n* = 3). ^∗^*p* < 0.05, ^∗∗^*p* < 0.01.

The release behavior of PTX from polymeric micelles was evaluated under various conditions at 37°C. Different buffer solutions were employed to simulate the micro-environment of the blood circulation (pH 7.4), tumor tissue (pH 6.5), and endosome (pH 5.5). We first studied the performance of pH-sensitive PEG-BHyd-dC_12_/PTX micelles. At pH 7.4, almost no PTX was released in the initial 4 h, which was followed by a sustained release phase with only 38% PTX release after 48 h (Figure 5A, black trace). Therefore, the micelles can stably encapsulate PTX for a long time, which is important for decreasing the side effects and increasing the drug accumulation in tumor sites. By lowering the pH to 6.5, a notable increase in drug release was observed at each time point (Figure 5A, blue trace). With further decrease of the pH to 5.5, the micelles showed an even higher rate of drug release (Figure 5A, red trace). After 48 h, the cumulative drug release was 50% and 65%, respectively, significantly higher than that at pH 7.4 (∼40%), indicating a good pH-responsive capability. This pH-responsive drug release profile can be ascribed to the hydrazone bond between the hydrophilic and hydrophobic chains of the polymer. As the pH decreases, the hydrazone bond tends to hydrolyse and thus the micelles collapse, resulting in burst drug release.

As a control, we also performed the drug release experiment with pH-insensitive PEG-BAmi-dC_12_/PTX micelles. In this case, slow and sustained drug release was seen under different conditions, and pH had little effect on the rate of drug release, giving a cumulative drug release of less than 40% after 48 h (Figure 5B). Considering the structural difference between PEG-BHyd-dC_12_/PTX and PEG-BAmi-dC_12_/PTX, these results further demonstrated critical role of the hydrazone bond for the pH-sensitive property of the PEG-BHyd-dC_12_/PTX micelles.

### Intracellular Uptake Study

**FIGURE 6:**
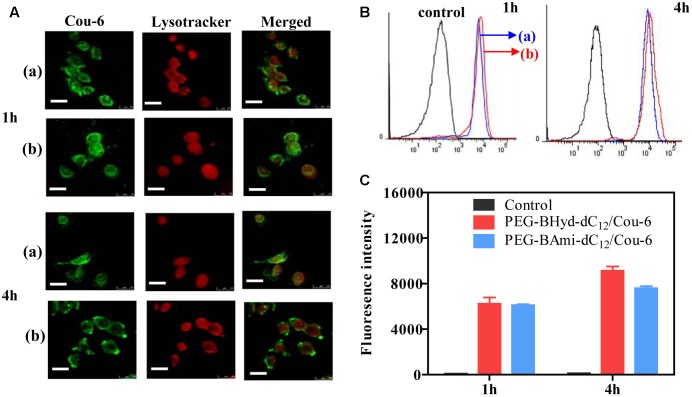
Cellular uptake studies of PEG-BHyd-dC_12_/Cou-6 and PEG-BAmi-dC_12_/Cou-6 in A549 cells by using CLSM **(A)**, flow cytometry **(B)**, fluorescence intensities quantified from **B** **(C)**. The (a) indicated PEG-BAmi-dC12/Cou-6 while the (b) represented PEG-BHyd-dC_12_/Cou-6. The scale bar is 25 μm.

Having demonstrated the pH-responsive property of the PEG-BAmi-dC_12_/PTX micelles, we next studied the performance of the micelles inside cells. To conveniently track the micelles inside cells, Cou-6 (a hydrophobic green fluorophore) instead of PTX was encapsulated into micelles, and the acidic organelles (i.e., lysosomes and endosomes) were stained by Lysotracker red. A549 cancer cell line was used as a model since PTX has been widely used in clinic for lung cancer therapy (Singla et al., 2002). From confocal laser scanning microscopy (CLSM), substantial green fluorescence was observed for both types of micelles after 1 h incubation (Figure 6A), indicating a high level of cellular internalization. To visualize the co-localization of micelles and endo/lysosomes, we merged the green and red channels, and the emergence of orange spots indicated the localization of micelles in the endo/lysosomes. Both PEG-BHyd-dC_12_/Cou-6 and PEG-BAmi-dC_12_/Cou-6 micelles showed obvious spots after 1 h of incubation, consistent with the endocytosis pathway of the micelles (Zhang et al., 2017).

We next studied the intracellular performance of the micelles. To do this, the cells were washed and cultured in fresh media so that further internalization of micelles was avoided. After 4 h incubation, the pH-insensitive micelles were still largely entrapped into the endo/lysosomes. In contrast, the orange spots of pH-sensitive micelles were weakened, and green color was evenly distributed throughout the cytoplasm, which showed minimal co-localization with the red fluorescence of the endo/lysosomes. The micelles detached from endosome due to hydrolysis of copolymer under acidic organelles, which facilitated efficient release of drug. Therefore, successful endo/lysosomal escape of pH-sensitive micelles was indicated. It is known that the successful escape of a nano-delivery system from the intracellular endosome/lysosome for drug release is a key issue in determining their therapeutic efficiency (Qiu et al., 2017). After cellular uptake, micelles were first entrapped into endosome/lysosome (Chou et al., 2011; Varkouhi et al., 2011). Once entering the endo/lysosomes, the pH-sensitive micelles were disassembled because of pH-triggered hydrolysis of the acid-labile chemical linkage, and the drug rapidly escaped from the endosome/lysosome, resulting in pH-triggered intracellular burst release (Fang et al., 2016).

To have a quantitative understanding, we next performed flow cytometry experiments to study the uptake of micelles by A549 cells (Figures 6B,C). After 1 h incubation, there was no difference in intensity between pH-sensitive and pH-insensitive micelles. Interestingly, after 4 h, the fluorescence from pH-responsive micelles was considerably higher than that of pH-insensitive micelles (Figure 6C), in agreement with a previous report (Qiu et al., 2017). While the pH-responsiveness of micelles has little effect on cell uptake process, the relative lower fluorescence for PEG-BAmi-dC_12_/PTX was likely due to the efflux of the micelles from cells to medium. As has been demonstrated, the endo/lysosome entrapped micelles can be pumped out by ATP-binding cassette protein B1 (ABCB1) transporter (Sakai-Kato et al., 2012). Since the pH-responsive micelles collapse faster in endo/lysosome, relatively less micelles were cleared from cells by this pump-out process, resulting in stronger fluorescence inside cells.

### Cytotoxicity Assay

**FIGURE 7:**
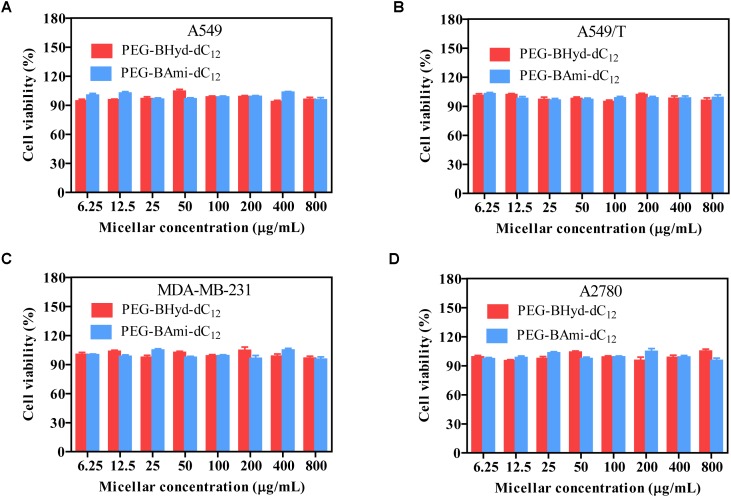
Cell viability of blank micelles after incubating with A549 **(A)**, A549/T **(B)**, MDA-MB-231 **(C)**, and A2780 **(D)** cells for 72 h. Data were shown as mean ± SD (*n* = 4).

**FIGURE 8:**
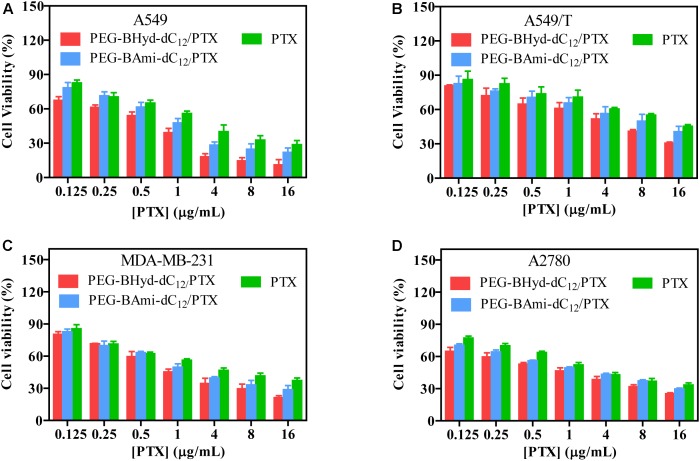
Cell viability of PTX-loaded micelles and free PTX after incubating with A549 **(A)**, A549/T **(B)**, MDA-MB-231 **(C)**, and A2780 **(D)** cells for 72 h. Data were shown as mean ± SD (*n* = 4).

Cytotoxicity studies were performed by incubating micelles with different types of cells for 72 h, and cell viability was measured by MTT assay. The cytotoxicity of the polymers was tested by incubating the cells with blank micelles (without PTX loading), and all types of cells remained >90% viability with concentration up to 800 μg/mL, indicating high biocompatibility (Figure 7). As for A549, at the highest PTX concentration (16 μg/mL), the viabilities of cells incubated with PEG-BHyd-dC_12_/PTX, PEG-BAmi-dC_12_/PTX and free PTX dropped to 11%, 22%, and 28%, respectively, showing high toxicity to cancer cells (Figure 8A). The anti-cancer capability was quantified by measuring the half-maximal inhibitory concentration (IC_50_), which was in order of PEG-BHyd-dC_12_/PTX (0.57 μg/mL) < PEG-BAmi-dC_12_/PTX (1.1 μg/mL) < free PTX (1.87 μg/mL) (Table 1). Therefore, PEG-BHyd-dC_12_/PTX exhibited the highest activity, which was attributable to the pH-responsive property for rapid endo/lysosome drug escape to enhance the antitumor effect.

**Table 1:** IC_50_ value of the micelles and free PTX to A549, A549/T, MDA-MB-231, and A2780 cells for 72 h incubation (mean ± SD, *n* = 4).

	IC_50_ (μg/mL)			
	A549	A549/T	MDB-MA-231	A2780
PTX	1.87 ± 0.08	11.17 ± 1.15	2.99 ± 0.37	2.01 ± 0.04
PEG-BHyd-dC_12_/PTX	0.57 ± 0.16^∗▲^	3.04 ± 1.13^∗▲^	1.16 ± 0.06^∗▲^	0.75 ± 0.08^∗∗▲^
PEG-BAmi-dC_12_/PTX	1.10 ± 0.06^#^	6.77 ± 0.30^#^	1.64 ± 0.13^#^	1.33 ± 0.13^#^

*PTX vs. PEG-BHyd-dC_12_/PTX, ^∗^p < 0.05, ^∗∗^p < 0.01; PTX vs. PEG-BAmi-dC_12_/PTX, ^#^p < 0.05; PEG-BHyd-dC_12_/PTX vs. PEG-BAmi-dC_12_/PTX, ^▲^p < 0.05.*

To test the generality, we further performed the anti-tumor assay by using MDA-MB-231 and A2780 cells, and analogous results were observed (Figures 8C,D). The PEG-BHyd-dC_12_/PTX displayed the best anti-cancer activity, followed by PEG-BAmi-dC_12_/PTX and then free PTX. Therefore, such micelles can be implemented for different types of cancer therapy. As one limitation of PTX for long-term cancer treatment is the acquired drug resistance by cancer cells (Yusuf et al., 2003), we also tested whether the nano-systems could reverse drug resistance by using PTX-resistant A549/T cells as a proof-of-concept. The cytotoxicity of PTX and micelles was also dose dependent (Figure 8B), while the overall IC_50_ value was much higher due to the drug resistance (Table 1). Notably, cytotoxicity of PEG-BHyd-dC_12_/PTX was 3.7-fold higher than that of free PTX, which may be useful to reverse drug resistance.

### Hemolysis Assay

**FIGURE 9:**
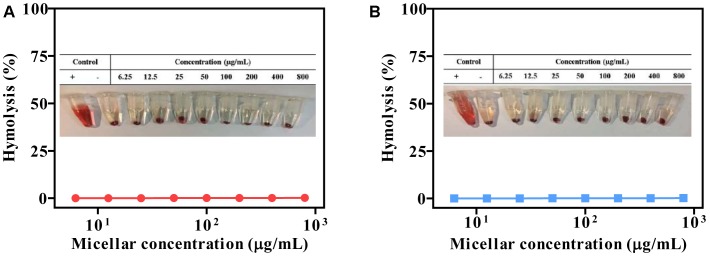
Compatibility studies of micelles (pH 7.4). PEG-BHyd-dC12 **(A)** and PEG-BAmi-dC12 **(B)**. “+” represents positive control by using 0.5% Triton X-100, and “–” represents negative control of non-treatment.

The biocompatibility of polymeric micelles is the prerequisite for biomedical application. We studied this property by using hemolysis assay. Typically, the micelles were incubated with erythrocytes, and the release of hemoglobin was measured to quantify the erythrocyte-damaging properties (Nogueira et al., 2013). The positive control of 0.5% Triton X-100 showed obvious hemolysis, as high as 100%, while the micelles produced less than 2% at different concentration (Figure 9). Therefore, the micelles were highly biocompatible and can be directly administrated by intravenous injection.

## Conclusion

In this work, pH-sensitive PTX-loaded PEG-BHyd-dC_12_ micelles were constructed and characterized. These nanoparticles exhibited pH-dependent drug release profile and endosomal escape ability after intracellular delivery, and displayed enhanced anti-tumor activity compared with the pH-insensitive counterpart micelles and the free PTX. All of these results suggested that the PEG-BHyd-dC_12_ micelles-based drug delivery system is a promising drug carrier for targeted cancer treatment.

## Author Contributions

YY performed the cell experiments and wrote the manuscript. ZW performed the synthesis and characterization. YP performed drug release. JD and WZ designed the experiments.

## Conflict of Interest Statement

The authors declare that the research was conducted in the absence of any commercial or financial relationships that could be construed as a potential conflict of interest.
